# Advances in 3D bioprinting technology for functional corneal reconstruction and regeneration

**DOI:** 10.3389/fbioe.2022.1065460

**Published:** 2023-01-06

**Authors:** Shuo Jia, Yashan Bu, Dzi-Shing Aaron Lau, Zhizhen Lin, Tianhao Sun, Weijia William Lu, Sheng Lu, Changshun Ruan, Cheuk-Hung Jonathan Chan

**Affiliations:** ^1^ Department of Ophthalmology, LKS Faculty of Medicine, University of Hong Kong, Hong Kong, Hong Kong SAR, China; ^2^ Department of Orthopedic and Traumatology, LKS Faculty of Medicine, University of Hong Kong, Hong Kong, Hong Kong SAR, China; ^3^ Shenzhen Gangqing Biomedical Technology Co. Ltd, Shenzhen, China; ^4^ Research Center for Human Tissues and Organs Degeneration, Institute of Biomedicine and Biotechnology, Shenzhen Institute of Advanced Technology, Chinese Academy of Sciences, Shenzhen, China; ^5^ Department of Orthopedic Surgery, The First People’s Hospital of Yunnan Province, Kunming, China

**Keywords:** 3D bioprinting, cornea, keratoprosthesis, reconstruction, regeneration

## Abstract

Corneal transplantation constitutes one of the major treatments in severe cases of corneal diseases. The lack of cornea donors as well as other limitations of corneal transplantation necessitate the development of artificial corneal substitutes. Biosynthetic cornea model using 3D printing technique is promising to generate artificial corneal structure that can resemble the structure of the native human cornea and is applicable for regenerative medicine. Research on bioprinting artificial cornea has raised interest into the wide range of materials and cells that can be utilized as bioinks for optimal clarity, biocompatibility, and tectonic strength. With continued advances in biomaterials science and printing technology, it is believed that bioprinted cornea will eventually achieve a level of clinical functionality and practicality as to replace donated corneal tissues, with their associated limitations such as limited or unsteady supply, and possible infectious disease transmission. Here, we review the literature on bioprinting strategies, 3D corneal modelling, material options, and cellularization strategies in relation to keratoprosthesis design. The progress, limitations and expectations of recent cases of 3D bioprinting of artifial cornea are discussed. An outlook on the rise of 3D bioprinting in corneal reconstruction and regeneration is provided.

## 1 Introduction

The cornea, the transparent outer wall of the anterior eyeball, has three major functions, which are; protective role as “shield”, light transmitting role as “window”, and refractive role as “focusing lens”. In corneal diseases, these three functions of the cornea can become compromised, as if presense of scarring (Dekaris et al., 2005) by stromal injuries (Lagali, 2020) or edema (Zhang et al., 2019a) from endothelial dysfunction (Shen et al., 2017). According to *World Report on Vision* released by WHO in 2019, at least 4.2 million people live with vision impairment secondary to corneal diseases (World Health Organization, 2019). Unfortunately, obtaining an autograft (equivalent tissue from the same eye or contralateral eye of the same patient) is usually not possible for replacement of cornea, therefore any further interventions to regain or improve vision are mostly dependent on cornea donation (Tan et al., 2012). What’s more, despite the fact that keratoplasty has been considered as having high long-term success rates with little requirement for systemic or lifelong immunosuppression, various risk factors such as medical history with inflammatory and ocular disease are still associated with high graft-failure rates, which aggaravates the demands of donor allografts (Armitage et al., 2019). One survey revealed that 15%–20% of patients expecting corneal transplantation remain untreated due to the shortage of corneal donors (Whitcher et al., 2001). Meanwhile, as accessibility of corneal allografts remains low due to their relatively high cost and inconveniences regarding the safe extraction, storage, and transportation of living tissue, 53% of the global population has no access to corneal transplantation (Gain et al., 2016; Ludwig et al., 2018). Since the worldwide supply is frequently inadequate in satisfying the demands of eradicating corneal blindness, a number of synthetic cornea replacement devices such as artificial corneas are actively being persued worldwide and have been approved on routine clinical practice over the past three decades (Brunette et al., 2017).

**FIGURE 1:**
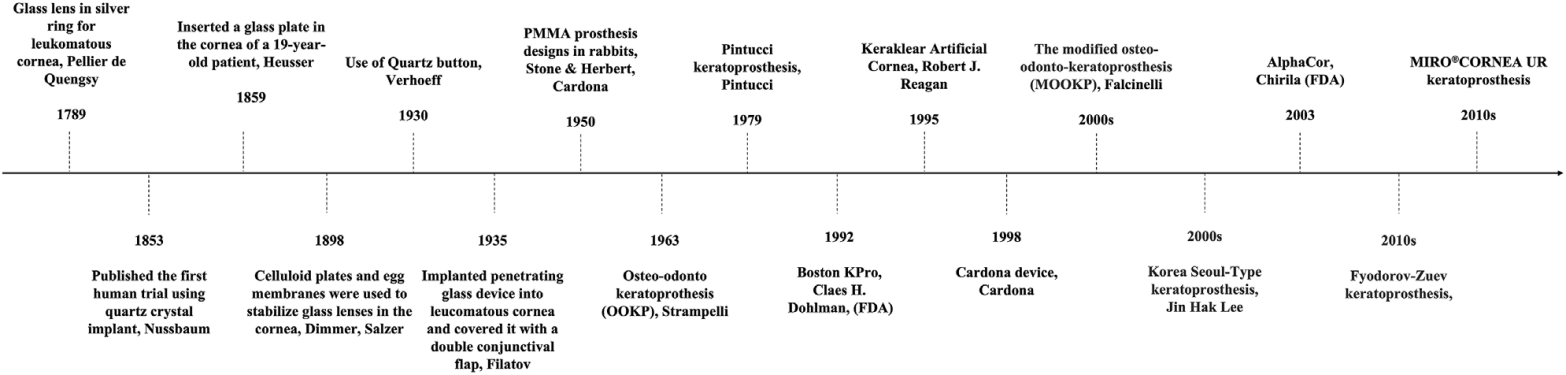
Milestones of corneal transplant including keratoprosthesis development.

**FIGURE 2:**
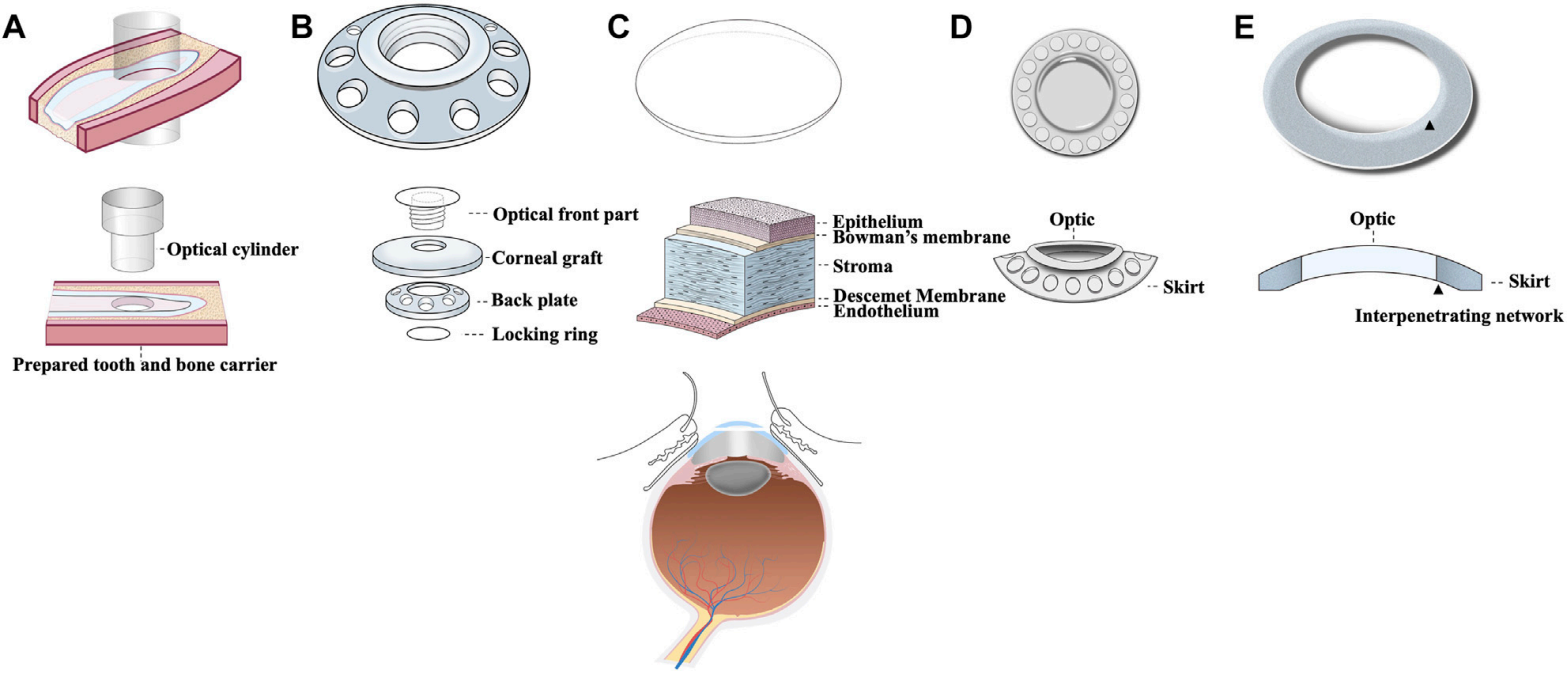
Schematic images of 5 commonly used corneal transplants including **(A)** OOKP, **(B)** Boston KPro, **(C)** Donor graft, **(D)** Keraclear, and **(E)** AlphaCor.

Artificial cornea is termed as keratoprosthesis where “kerato” and “prosthesis” are from the greek word “cornea” and “addition” respectively (Dalisay, 2016). It was a French ophthalmologist Pellier de Quengsy from Montpellier, France who is traditionally considered to be the first one to describe “artificial cornea” in 1789 (Cortina and de la Cruz, 2015). Details of the historical background of keratoprosthesis are described in Figure 1. Current keratoprosthesis mostly depends on the structural combination of a stable optical cylinder with a connective periphery that can integrate with host corneal tissue (Griffith et al., 2008). The connective periphery uses low optical quality donor peripheral cornea or porous bio-affinitive material like titanium and Poly (hydroxyethyl methacrylate) (PHEMA). Schematic images of four types of commonly used keratoprosthesis are displayed in Figure 2. Despite these measures, none of the approved keratoprosthesis to date is yet ideal as a replacement of cornea.

**FIGURE 3:**
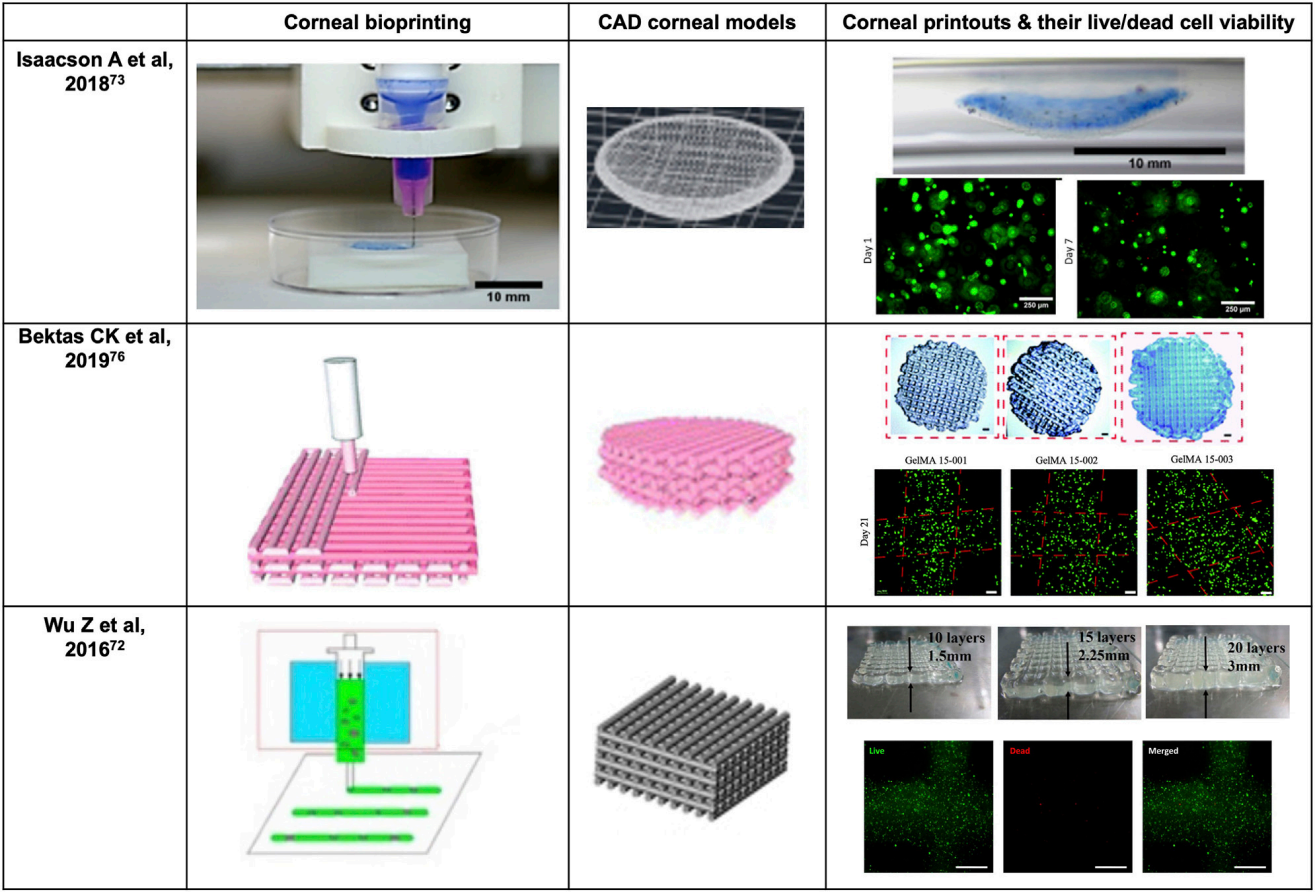
Representative progress on 3D bioprinting of cornea.

Three-dimensional (3D) bioprinting allows scientists to print objects into desired steric shapes with high resolution and diversified patterns (Zema et al., 2017). Unlike conventional tissue engineering, bioprinting supports the precise deposition of bio-inks in a prescribed pattern corresponding to the organotypic anatomic cues, thereby potentially offering the advantages of personalization of refractive power, complex multi-layer structure, and spatial heterogeneities (Haring et al., 2017). 3D bioprinting techniques, preprinting modelling and design, and specific mechanical strength and biocompatibility of the bioinks used all contribute to the successful biofabrication of a personalized cornea. Herein, as summarized in Table 1 and Table 2, the literature on bioprinting methods, corneal 3D modelling, material options, and cellularization strategies in relation to keratoprostheses design were reviewed in hope of providing the researchers prospectives in 3D bioprinting of functional cornea (Figure 3).

**TABLE 1:** Summary of original studies on 3D bioprinting of cornea.

Publication details	Type of study	Bioprinting techniques	Preparation of digital cornea model	Biomaterials	Incorporation of cellular components	Main findings and results
Epithelial layer						
Wu et al. (2016)	*in-vitro*	Extrusion-based bioprinting	CAD; plain	Sodium alginate and gelatin powder (type A) & neutralized rat-tail type I collagen	Human corneal epithelial cell line	Controllable degradation of the alginate matrix with the help of sodium citrate
Zhang et al. (2019b)	*in-vitro*	DLP printing and extrusion-based printing	CAM; Pentacam images	Sodium alginate and gelatin	Human corneal epithelial cell line	Geometry-controllable corneal substitutes
Stromal layer						
Isaacson et al. (2018)	*in-vitro*	Extrusion-based printing (INKREDIBLE printer, CELLINK AB)	CAM (AutoCAD 2017); rotating Scheimpflug images	Sodium alginate and methacrylated type I collagen	Primary human corneal stromal cells	human corneal substitutes fabrication aiming at clinical suitability
Campos et al. (2019)	*in-vitro*	Electromagnetic micro-valve bioprinting	CAD; Dome-shaped	Type I collagen and agarose	Primary human corneal stromal keratocytes	Freeform and cell-friendly drop-on-demand technique to fabricate translucent corneal stromal equivalents with optical properties like real corneal stromal tissue
Bektas and Hasirci, (2020)	*in-vitro*	Extrusion-based bioprinting (Bioscaffolder, SYS-ENG)	CAD (Sketchup); plain	GelMA	Primary human corneal keratocytes	3D printed HK seeded corneal stroma with synthesis of the specific collagens and proteoglycan by the seeded keratocytes
Endothelial layer						
Kim et al. (2019)	*in-vitro, in-vivo and ex-vivo*	Extrusion-based bioprinting (EDISON INVIVO, ROKIT)	CAD; plain	Gelatin and arginylglycylaspartic acid (Arg-Gly-Asp; RGD); commercial lyophilized bovine AM	Primary human corneal endothelial cells transfected with RNase 5 siRNA	3D bioprinted R5-hCEC-laden AM endothelial grafts in a descemetorhexis-induced corneal endothelial decompensation model in rabbits
Epithelial + stromal layers						
Sorkio et al. (2018)	*in-vitro*	Laser-assisted bioprinting	CAD; plain	Laminin-521 and serum-free CnT-30 medium and Hyaluronic acid sodium salt; Human Collagen Type I & EDTA human female AB blood plasma & Thrombin from human plasma	Primary human adipose derived stem cells and Human embryonic stem cells derived limbal epithelial stem cell line	Layered 3D LaBP bioprinted tissues using human stem cells and human protein based bio-inks
He et al. (2022)	*in-vitro and in-vivo*	DLP bioprinting	CAD; plain	20% PEGDA & 5% GelMA	Rabbit corneal epithelial cell lines and rabbit adipose-derived mesenchymal stem cells	DLP bio-printing corneal epithelium/stroma bilayer
Epithelial + stromal + endothelial layers						
Zhang et al. (2017)	*in-vitro and in-vivo*	DLP bioprinting	CAD; plain	7.5% GelMA with 2.5% HAMA (200 kDa); 7.5% Acryloyl-collagen with 25% PEGDA (700 kDa) based matrix	Primary human corneal stromal cells, limbal stem cell differentiated corneal epithelial and endothelial cells	Corneal reconstruction based on the multi-laminar anatomy of cornea
Acellular scaffold						
Kutlehria et al. (2020)	*in-vitro*	SLA printing (SLA printer, FORMLABS) & extrusion-based printing (BIO X 3D printer, CELLINK)	CAD (Autodesk Fusion 360); Central thickness of 500 μm, periphery thickness of 700 μm, unified radius of 5.80 mm, and a sagittal height of 3.0 mm	Sodium alginate (MW 50 kDa) and gelatin Type B from bovine skin (MW 50–100 kDa) and Type I bovine collagen	N	High throughput/rapid printing on large scale
Kong et al. (2020)	*in-vitro and in-vivo*	Direct writing (custom-made)	CAD; plain	Poly (ε-caprolactone)-poly (ethylene glycol) & GelMA	N	3D fiber hydrogel construct and serum-free media synergize maintains keratocyte phenotype

**TABLE 2:** Summary of different types of 3D printing technology and their applications.

Techniques	Type of 3D bioprinting	Advantage	Drawback	Applications
3D hydrogel-based cell-laden technique	Extrusion bioprinting technology	Better cellular compatibility and increased robustness of the construct	Prominent strip-like patterns undesirable for transparent; relatively poor resolution; inadequate stiffness required for precise tailoring of the desired 3D shape	HCEC and 3D-engineered corneal epithelium Wu et al. (2016)
the Freeform Reversible Embedding of Suspended Hydrogels (FRESH) method87 with low viscosity bio-inks	Pneumatic extrusion bioprinting	Able to accommodate material viscosities as low as 30 mPa/s		Keratocyte-laden corneal stromal equivalents Zhang et al. (2019b)
Laser-induced-forward-transfer (LIFT) technique Sorkio et al. (2018)	Laser-assisted bioprinting	Better resolution down to micron/nano-scale	Slow and theoretically poor continuity of printed material	Human stem cell-based structure mimicking corneal tissue Sorkio et al. (2018)
Drop‐on‐demand (DoD) inkjet bioprinting strategy (Campos et al., 2019) & aerosol jet printing (AJP) techiques (Gibney et al., 2021)	Liquid spreading or inkjet printing	best for printing low viscosious tissues or materials	Imprecise application	Human cornea Zhang et al. (2019b) and human collagen Gibney et al. (2021)
Combined DLP and extrusion bioprintings	Digital light processing (DLP) printing	Higher efficiency high precision	Less ideal resolution for creating constructs with curvature	3D printed cornea Zhang et al. (2019b); He et al. (2022)

## 2 Challenges in current keratoplasty

Despite successful treatment of the initial corneal disease or ocular condition (such as corneal ulcer and trachoma), the affected eye is often associated with residual corneal scarring and opacity, hence corneal transplantation is imperative to fully recover a clear vision (Wilson et al., 2012). In 1905, the first human corneal transplantation was performed by an Austrian ophthalmologists, Eduard Zirm, in Moravia (present-day Czech Republic) (Maienschein, 2011; Flockerzi et al., 2018). With striking advances in surgical techniques over the recent decades, cornea has become the most commonly transplanted tissue worldwide (Tan et al., 2012). Corneal transplantations are divided into two main categories: penetrating keratoplasty (PKP) and lamellar keratoplasty (LK) (Akanda et al., 2015). In PKP, the entire thickness of cornea is displaced by donor grafts (Akanda et al., 2015). While in LK, which is more recently developed, merely the corneal lesions are replaced with donor equivalents leaving the healthy areas untouched (Mobaraki et al., 2019).

The immune privilege is beneficial for protecting transplanted cornea from damaging effects of excessive immune-mediated inflammatory response thanks to i) existence of blood-ocular barrier without lymphatic drainage that minimizes antigens’ entry into the regional lymph nodes or foreign macromolecules into the eye; ii) lack of antigen-presenting cells and quiescence of immune-competent cells; iii) expression of immunosuppressive factors such as TGF-β and alpha-melanocyte-stimulating hormone (α-MSH) in anterior segments; iv) existence of FasL- or TRAIL-induced apoptosis that eliminate the activated inflammatory cells. However, considering the limitations in transplantation surgery itself, poor long-term outcomes such as graft rejection or late graft failure remains to be a challenge, despite the relative immune privilege of the cornea and anterior chamber (Coster and Williams, 2005). In addition, mostly restricted to situations where the chance of failure with a donor allograft is high, keratoprosthesis is frequently facing an indolent inflammatory reaction to the prosthetic material (Gomaa et al., 2010). Stulting et al. reported 23% of keratoplasty patients experienced at least one rejection event within 5 years post-op, with risk factors for rejection that include pseudophakic or aphakic corneal edema and female gender (Stulting et al., 2012). In particular, the vertical stromal wound resulted from penetrating keratoplasty (PKP) may lead to delayed wound healing that does not only create unpredictable and shifting refractive error for the patients, but also increases the risk of late incision rupture and loss of the eye (Binder et al., 1975; Perry and Donnenfeld, 1988; Rehany and Rumelt, 1998; Tseng et al., 1999; Abou-Jaoude et al., 2002; Lam et al., 2007). Furthermore, the sutures of PKP makes the cornea susceptible to infection, ulceration, vascularization, rejection, and unpredictable astigmatism of varying magnitude, despite of diverse ingenious suturing techniques (Confino and Brown, 1985; Reddy et al., 1987; Binder, 1988; Belmont et al., 1993; Serdarevic et al., 1995; Gross et al., 1997; Davis et al., 1998; Riddle et al., 1998; Akova et al., 1999; Ruhswurm et al., 1999; Seitz et al., 1999; Stechschulte and Azar, 2000; Jonas et al., 2002). These drawbacks suggest the potential benefit to update graft alternatives and incoperate tissue engineering for improved liabilities in optics maintainance and wound healing. Fortunately, as a collaborative effort between ophthalmalogists, bio-engineerers, and materials scientists, novel development of keratoprosthesis makes possible the use of biomaterials with tunable performances and the incorporation of a transplant recipient’s own cells in the engineered tissue, which endows higher resistance to immune rejection or other clinical complications.

## 3 Corneal tissue engineering

Tissue engineering applies to the creation of biological or semi-synthetic living organs for repairing, restoring, and regenerating human tissue anatomically and functionally (Dash et al., 2011). Given the immune privilege and avascular nature, cornea is an anatomically and physiologically attractive organ type in tissue engineeering (Fuest et al., 2020). Aiming to imitate natural corneal characteristics, three primary sources including cellular components, growth factors, and biomaterials to fabricate the biomedical parts contributes equally on corneal tissue engineering (Chen and Liu, 2016). For example, it has been reported that the keratocytes differentiated from human corneal stem cells (hCSCs) could secret multiple layer of orthogonally-oriented collagen fibrils with supplementation of FGF-2 (10 ng/ml) and TGF-β3 (.1 ng/ml) (Maiti et al., 2021).

Tissue‐engineered corneas offer certain advantages over donor corneas, including: i) no need for individual donor health screening, ii) mass production is possible, iii) tailored requirement on biomechanical, optical, or biological characteristics of keratoprostheses for personalized medicine is now feasible (Mitsuishi et al., 2013). As one of the rapidly emerging assembly methods of tissue engineering, 3D printing makes possible constructing outputs with sophisticated geometric patterns by depositing materials based on the digital commands (Zhang et al., 2020). Therefore, it is anticipated that advancing 3D bioprinting technology will make it possible to achieve controllable corneal curvature and thickness according to individualized patients’ refractive needs, and to tackle the disadvantage of “one size fits all” in traditional tissue engineering (Naboni and Paoletti, 2015). Although corneal 3D printing only arose in the last 5 years, multitudinous 3D bioprinting techniques are available now targeting the corneal lesions.

## 4 Emphasis on corneal functional reconstruction

As the first function of keratoprosthesis is to allow the passing of light into the eyeball and onto the optic nerves, where electrophysiological impulses are sent to the central nerve system (CNS), the waterlike clarity is therefore desired (Sayegh et al., 2010). This fact turns the focus to materials including glass, plastics or hydrogel (Myung et al., 2008). Initially recommended by Wichterle and Lím in 1960, a hydrogel is a stereoscopic three-dimensional (3D) network of hydrophilic polymers capable of absorbing and holding plenty of water but retaining the shape by chemical or physical cross-linking of individual polymer chains (Bahram et al., 2016). The extent that water could be held within the polymers determines not only the light transmittance ability but also the permeability of small molecule nutrients such as glucose, or albumin, etc. (Kashyap et al., 2005) For instance, Alphacor, an FDA-approved device made of Poly (hydroxyethyl methacrylate) (PHEMA) optics and sponge skirts, and the most recent version of BiokproII made by poly tetrafluoroethylene (PTFE) skirt and a central optic poly vinyl pyrrolidone (PVP)-coated polydimethylsiloxane (PDMS) silicone rubber are both hydrogel products with nice optical clarity. However, though inheriting the hydrophilic nature of hydrogel, both devices face similar problem of clinical complications such as stromal melt or long term calcification due to unsatisfying nutrient permeability (Hicks et al., 2005; Holland et al., 2021). Hence, hydrogel with competitive hydrophilicity as well as sufficient equilibrium water content is necessary for corneal products including contact lens and corneal alternatives (McGlinchey et al., 2008).

The second function of keratoprosthesis is shielding the eye from infection and foreign material, an ideal keratoprosthesis is assumed to hold tensile strength similar to or better than the natural corneal tissue, more specifically, strong enough to suit the surgical manipulation and fixation of PKP (Wnek and Bowlin, 2008). Failure in sufficient strength results in an open communication between external environment and the inside of the eye, which will almost always mean blindness from severe hypotony or endophthalmitis (Avadhanam et al., 2015). Therefore, the strength level of the keratoprosthesis must allow the eyeball to be sealed, both to keep bacteria or toxins outside and aqueous humor inside (Robert and Dohlman, 2014). As a safer alternative to full thickness transplant, though reserving the posterior lamellae, LK graft is not exclusively expected to serve a tectonic purpose and provide structural strength to the cornea after the defective area (e.g., with scarring) has been removed (Wilkie and Whittaker, 1997). Apart from tensile strength which supports the fixation, incorporation of the material into the surrounding host cornea, i.e., capability of biointegration, contributes to the fixed positioning of the graft avoiding any loosening or dislodge (Anderson et al., 1996). In order to improve biocompatibility and clinical outcome, advanced biomaterial such as collagen, gelatin and Gelatin methacryloyl (GelMA) are adopted in ongoing research for the fabrication of newer keratoprosthesis prototypes (Bajaj et al., 2014). Nevertheless, none of them has achieved balance between the mechanical properties and bioaffinity.

The third function of cornea is refracting and bending light to be perfectly sensed by the rods and cones that detect light in the retina of the eye (Bruce et al., 2003). In order to achieve the refractive function, again the structural stability owned by sufficient tensile strength of the material is quite essential, without which not only the maintainance of the patients’ personalized refractive power, but the safety or the related biocompatibility is challenged (Maluf and Williams, 2004). Actually, the dependence on structural stability explains why none of the commercially available keratoprosthesis are degradable (Alexander et al., 1996). Except that in clinical setting, for eyes with severe ocular surface disease or epithelial defect that will not heal despite frequent lubricants, amniotic membrane may be adopted to cover the cornea (like a contact lens), which usually degrades after 1–2 weeks (Nubile et al., 2011). Nevertheless, it would be disastrous if the speed of degration exceeds that of the regeneration, where an adjustable degradation extent and speed are desirable for the successful postoperative outcome of keratoprosthesis transplantation. With the help of the highly stable material, the precise topography including keratometry and pachymetry would be achievable as long as the material could be efficiently and economically deposited (Ziaei et al., 2015).

## 5 Diverse bioprinting techniques

**FIGURE 4:**
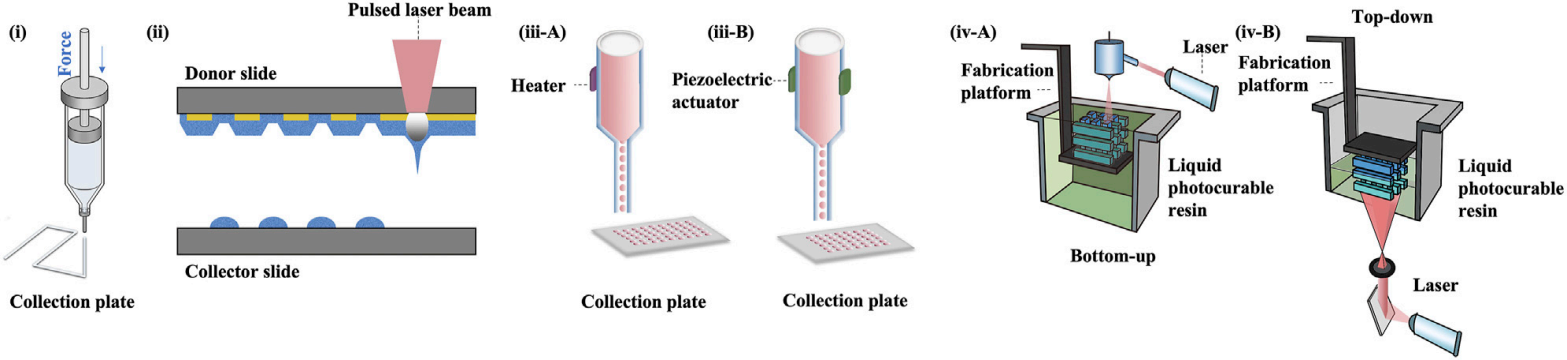
3D bioprinting technologies. **(i)** extrusion bioprinting, **(ii)** laser-assisted bioprinting, **(iii A-B)** inkjet bioprinting, **(iv A-B)** SLA and DLP bioprinting.

Since the introduction of bioprinting, the prevailing techniques have been constantly updated (Mota et al., 2020). A variety of commonly adopted 3D bioprinting techniques are schematically displayed in Figure 4.

Nevertheless, the conventional extrusion bioprinter is still preferred by many researchers (Schwab et al., 2020). Pneumatic extrusion-based bioprinting was shown to accommodate viscosities of the bio-inks as low as 30 mPa/s as driving by the air-pressure provided by the pump system (Sorkio et al., 2018). In 2016, Wu Z et al. reported hydrogel-based cell-encapsulated technique to bioprint 3D-engineered corneal epithelium. In this research work, an extrusion-based 3D cell-printing machine was developed for rapid prototyping of bioengineered organs (Wu et al., 2016). In 2018, Isaacson A et al. employed the extrusion-based bioprinter (INKREDIBLE; Cellink) to fabricate corneal stromal equivalent, the first such reported and a milestone in the field of corneal printing (Isaacson et al., 2018). In this study, keratocyte-laden corneal mimicry scaffolds were successfully bioprinted with specially engineered low viscosity bio-inks at high precision rates with the assistance of freeform reversible embedding of suspended hydrogels (FRESH) method (Zhang et al., 2019b). In this study, the organized alignment of collagen fibrils was found to contribute to bioactivity of the device. In 2019, Kim H et al. once again adopted extrusion-based printer for shear induction to the collagen fibrils alignment *via* the change of nozzle diameter and flow rate and successfully ameliorated the cellular events (Kim et al., 2019). More recently in 2020, Bektas CK et al. reported using low temperature dispense head of 3D bioprinter (BioScaffolder; Analytik) to produce GelMA hydrogels (Bektas and Hasirci, 2020). In the same year, Kutlehria S et al. used the bioprinter (BIO X; Cellink) for high-throughput automation of generating corneal stromal substitutes (Murphy and Docherty, 1992). Though offering unique advantages over conventional corneal engineering in the precise tailoring of structures, 3D pneumatic extrusion printing can result in prominent strip-like patterns, where layers are formed by linear extrusion, which would be undesirable for transparent constructs intended to replace corneal tissues (Isakov et al., 2016). The visible blank between the printed lines also leads to weak connection and sometimes opacities. In addition, the resolution of traditional extrusion is relatively poor even when using the thinnest nozzles and sometimes inaccurate due to shear thinning property (Kirillova et al., 2021). It is also difficult to retain the desired 3D shape precisely, unless the printed material is of adequate stiffness, or a base/support is utilized in case of low viscous bio-inks, e.g., using FRESH supports (Hinton et al., 2015).

In 2018, Sorkio A et al. employed laser-assisted bioprinting (LaBP) based on laser-induced-forward-transfer (LIFT) technique to construct human stem cell-based corneal mimicking scaffolds, with resolution down to micron/nanoscale (Sorkio et al., 2018). In 2020, Kong B et al. employed custom made Direct-Ink-Writing (DIW) device for the formation of the hybrid fiber hydrogel to induce the regeneration of corneal stroma (Kong et al., 2020). The millimeter-scale distance between the nozzle and collector of the direct writing technique improved the control over fibers and avoided the bending instability issue (Brown et al., 2014). Nevertheless, both laser-assisted and electron-assisted addictive manufacturing is slow and theoretically owns the poorest continuity of printed material due to the dot-by-dot build-up nature of these techniques but can achieve better resolution than pneumatic extrusion.

Other commonly used techniques include liquid spreading or inkjet printing, which can be limited by imprecise application, especially at the corners of the construct, but are best for low vicious tissues or materials (Gibson et al., 2021). In 2019, Campos DFD et al. adopted drop‐on‐demand (DoD) inkjet-based bioprinting strategy based on an electromagnetic micro‐valve for constructing 3D models mimicking human cornea (Campos et al., 2019). In 2021, aerosol jet printing techniques were recently introduced for bioprinting of human collagen by Gibney R et al. (Gibney et al., 2021) However, in bioprinting method such as the aerosol jet printing (AJP) system, the structure is usually denser compared to others (Gibney et al., 2021). For example, the average effective elastic modulus of AJP system printed recombinant human collagen type III (RHCIII) was 506 ± 173 kPa (Gibney et al., 2021). Nevertheless, an adoption of inkjet printing in building up epithelial or endothelial sheet with or without biomimicry artificial Bowman’s or Descemet’s layer would be becomingly considerable.

Stereolithography apparatus (SLA), the first type of light-assisted 3D printing to be introduced and still in use, is commonly limited in speed due to its dot-by-dot process but have advantages in the reconstruction continuity (Yu et al., 2020). Digital light processing printing (DLP), which came after SLA, is another popular light-assisted 3D printing technique that is faster since all dots of a layer are processed at once (del Barrio and Sánchez‐Somolinos, 2019). However, the resolution is still not ideal for creating constructs with curvature, since the curved surface often appears as steps or terraces even with resolution down to 10 μm. In 2018, Zhang B et al. utilized DLP printing for the supporting base used in determining the desired curvature for a 3D printed cornea (Zhang et al., 2019b). The concave supports of cornea is printed using the DLP module where stepwise patterns are clearly observed, while the convex object with individual thickness is bioprinted using the extrusion-based module (Zhang et al., 2019b). The combined DLP and extrusion bioprinting manufactures biosynthetic corneas with controllable geometric characteristics such as thickness and curvature which allows for high-precision management in corneal construction. Besides its efficacy in corneal bioprinting, this method also has the potential in fabrication of other shell-like or hollow structures with complex surfaces (Zhang et al., 2019b). In 2022, He B et al. used DLP-bioprinting to build biomimetic epithelium/stroma bilayer hydrogel implant for corneal regeneration (He et al., 2022). Though both CAD designed plain, and dome-shaped model were printed, the stepwise pattern resulting from the unsatisfying resolution and friability resulting from the mechanical properties of the biomaterial makes the printed dome-shaped hydrogel construct impossible for *in-vivo* models. Hence, CAD plain models for separately printing corneal epithelial and stromal layers were eventually adopted for rabbit experiment. As the resolution of the current DLP printer especially for printing 3D spatial models is not high, new printers to have better resolutions to print in finer details are highly expected. For example, future update on light-assisted 3D printing especially holographic laser projection, which is expected to have higher level of resolution, and greater ability to construct different replacement tissues using more organ-specific bio-inks with no doubt would populate as a trend for developing the main body, i.e., stromal component (Zhang et al., 2019b).

In summary, as with traditional extrusion printing, emerging bioprinting techniques also possess specific strengths and weaknesses (Carrow et al., 2015). Therefore, a combination of the special advantages of different bioprinting techniques in constructing various layers may contribute to developing an eventually desirable keratoprosthesis.

## 6 Computer aided modelling

3D printing, or additive manufacturing, is defined as constructing a stereoscopic object corresponding to a digital scanned organotypic or pre-designed model (Lichtenberger et al., 2018). In addition to providing structural strength and integrity to the eye along with the sclera, the cornea also possesses certain refractive characteristics that are essential for normal vision (Shimmura and Tsubota, 2006). In particular, the refractive characteristic of any synthetic cornea should be capable of individualization so that images can be precisely focused onto the retina in all cases, due to the wide variation in lenticular refractive power and axial length of human eyes in real world settings (Flitcroft, 2012). These specific characteristics necessitate greater complexity in the material properties and structural design of corneal replacement tissue compared to scleral replacement tissue. In 2018, Isaacson A et al. adopted a patient-specific individual corneal model based on a series of rotating Scheimpflug images (Isaacson et al., 2018). AutoCAD 2017 (version 20.1) was used by their team to seal the rim of the model cornea with a planar circle (*r* = 6.5 mm) for a dome-resembled model (Isaacson et al., 2018). In the next year, Zhang B et al. used a commercially available medical scanning instrument (Pentacam HR; OCULUS), for obtaining measurement data of a normal human cornea before applying these to computer aided modeling (Zhang et al., 2019b). In 2020, Kutlehria S et al. utilized corneal measurements of human adults and a digital modeling software (Fusion 360) to build a 3D stroma (Kutlehria et al., 2020). However as printing a curved structure is difficult and requires greater efforts, many researchers sacrificed the smooth curvature of the normal cornea when attempting bioprinting of the cornea. In 2019, Campos DFD et al. also utilized AutoCAD to create a dome-like model with diameter of 20 mm, height of 4 mm and thickness of .3 mm (Campos et al., 2019). In 2020, Bektas CK et al. directly used two-dimensional (2D) layer for printing and obtained a scaffold with decent mechanical strength and biocompatibility (Bektas and Hasirci, 2020). In 2022, He B et al. printed a 3D dome-shaped corneal scaffold and used 2D cylindrical structure for printing separate corneal epithelial and stromal scaffolds for *in-vitro* and *in-vivo* evaluation (Figure 6) (He et al., 2022).

It seems likely that for future corneal bioprinting, a patient-specific design that is precisely constructed using advanced computing and high-resolution bioprinters would be possible from a collaborative effort between ophthalmologists and bioengineers. In such situations, the need for subsequent secondary laser refractive surgery (e.g., LASIK) for the correction of unmet refractive power would be minimal as the 3D bioprinted cornea would be specifically tailored, in terms of shape and curvature, to the postoperative refractive aim desired by the patient (e.g., emmetropia) (Dai, 2008).

## 7 Materials

With the concept introduced 50 years ago, a biomaterial is a substance that has been engineered to interact with biological systems for a medical purpose, either a therapeutic (treat, augment, repair, or replace a tissue function of the body) or a diagnostic one (Williams, 2009). Materials ideal for constructing the artificial corneal using 3D bioprinting techniques should not only possess the mechanical strength of the natural corneal, but also with high biocompatibility, excellent optical clarity and ocular integrability, and with suitable interconnected pore structures. Despite challenges in finding out a bioink fulfilling all criteria, efforts have been made to improve and refine the 3D scaffold into a state closest to a natural cornea.

### 7.1 Mechanical properties

As the outer wall protecting intraocular components, the corneal shape constructed is expected to be stable regarding hardness, stiffness and elasticity (Pal, 2014). To fulfil these requirements, PMMA and PHEMA are the top common biomaterials that have been used for keratoprostheses but with the disadvantage of poor biocompatibility (Lloyd et al., 2001). As constructs using purely synthetic material, they tend to have low integration with the surrounding ocular tissues, especially hard to achieve epithelialization and may become extruded or dislodged unless held in place by some special means, including more complex suturing or tissue glues (Dee et al., 2003). However, sutures and glues are deemed to lose tension or adhesive strength with time, or the suture themselves can lead to local irritation and erosion (Khanlari and Dubé, 2013). Hence, it is challenging but critical to find an ideal material that has excellent optical clarity, sufficient mechanical strength, but with good biological ocular compatibility and integrability.

The biomechanics of the cornea are responsible for its functional responses and greatly impact vision. Of the natural cornea, it mainly stems from the structure of the collagen skeleton in the corneal stroma (Daxer et al., 1998; Dias and Ziebarth, 2013). In corneal engineering, mechanical characteristics are commonly indicated by elasticity, compression capability, and viscosity, etc., from the sample deformation extent in response to a particular mechanical load (Daxer et al., 1998). Dias JM et al. investigated the mechanical properties of the cornea stroma with the assistance of atomic force microscopy (Dias and Ziebarth, 2013). With human corneal samples collected from nine individuals, their results revealed that the Young’s modulus was 281 ± 214 kPa for the anterior stroma and 89.5 ± 46.1 kPa for the posterior stroma, meaning that the effective elasticity of the anterior lamellae consistently outstrips the posterior lamellae (Dias and Ziebarth, 2013). These results have demonstrated the presence of a biomechanical gradient of the stromal lamellaes (Dias and Ziebarth, 2013). Therefore, it would be insightful to consider designing multiple gradients of layers even in building up corneal stroma. Among varieties of biomaterials, hydrogels display distinct biotic mechanical trait in soft tissue replacement (Ahmed, 2015).

### 7.2 Hydrogels with higher biocompatibility

As a major component of corneal stroma, collagen is a natural choice of biomaterials for constructing bioengineered corneal structures (Cen et al., 2008). A major challenge with using collagen concerns the precise control of collagen concentration to achieve the necessary mechanical strength required when using an extrusion-based 3D bioprinter. To overcome this, composite bio-inks consisting of both collagen and alginate are preferred as they can bond the tensile strength of collagen with alginate and achieve appropriate printability (Levato et al., 2020). Alginate hydrogel is a popular biologically inert hydrophilic material that is widely used in 3D bioprinting, especially in extrusion-based printing (Jacob et al., 2022). Alginate is a naturally occurring anionic polymer typically obtained from brown seaweed and has been extensively investigated and used for many biomedical applications (Jacob et al., 2022), due to its biocompatibility, low toxicity, relatively low cost, and mild gelation by addition of divalent cations such as Ca^2+^. The highlight of Wu’s study in 2016 is that collagen was successfully extruded from the nozzles by mixing with a nice-printable gelatin/alginate system (Wu et al., 2016). The addition of alginate not only improved the imitation to ECM with better cellular compatibility, but also increased mechanical robustness of the construct with an intensified fibrous structure (Wu et al., 2016). In 2018, Isaacson A et al. also illustrated that the mechanical stability got enhanced with the gaining in the incorporated alginate portions and reach the highest at composition of one part 8 mg/ml collagen along with two parts alginates (Isaacson et al., 2018). Apart from alginate, agarose is another promising hydrogel biomaterial that can have high gel strength at low concentration (Afewerki et al., 2019). Agarose is generally extracted from certain red seaweed and is a linear polymer with a molecular weight of about 120,000, consisting of alternating D-galactose and 3,6-anhydro-L-galactopyranose linked by α-(1→3) and β-(1→4) glycosidic bonds (Afewerki et al., 2019). In 2019, Campos DFD et al. tried with .3% acidic type I collagen plus 3% agarose solutions (Campos et al., 2019). Gelatin or chemically crosslinked GelMA appears weak and brittle in terms of mechanical properties, thereby precluding from load bearing applications by themseves. But both gelatin and its derivative GelMA is cell-friendly and could facilitate the regeneration of ECM. In 2018, Zhang B et al. employed .10 g/ml gelatin and .02 g/ml sodium alginate in extrusion-based bioprinting of corneal stroma and adopted GelMA solution in DLP 3D printing of the supporting base (Zhang et al., 2019b). In 2020, Bektas CK et al. 3D printed with 15% GelMA solution in HK medium (Bektas and Hasirci, 2020). Also, in 2020, Kutlehria S et al. made their bio-inks by mixing sodium alginate, type B gelatin, and type I bovine collagen (Kutlehria et al., 2020). Other than collagen and alginate, natural polymers including hyaluronic acid, chitosan, etc. also have a wide application in constituting corneal mimicking scaffold. However, most natural polymers have a narrow MW range for achieving tunable mechanical or watery properties and can induce an immune response *in vitro* or *in vivo* (Liang et al., 2020). In addition, the disadvantage of using biocompatible but degradable material like collagen and GelMA is their dependence on the host tissue or organ to adequately regenerate extracellular matrices before they lose their mechanical properties (or is completely degraded), which is totally unacceptable in PKP, and is commonly accompanied by excessive activation of fibroblasts with subsequent scarring (Annabi et al., 2014). This is especially disadvantageous for corneal stromal replacement due to the need for media clarity to achieve optical success. Moreover, ideally, biocompatible materials possessing both optical transparency and stable structural strength are preferred as bio-inks for 3D bioprinting of synthetic corneal stromal tissue (Kim et al., 2020). Considering this, in 2021, Kong et al. fabricated grid poly (ε-caprolactone)-poly (ethylene glycol) microfibrous scaffold and infused the scaffold with gelatin methacrylate (GelMA) hydrogel to obtain a 3D fiber hydrogel construct (Kong et al., 2020). The combination of hydrogel and Grid-like PECL microfibrous scaffolds can reinforce the strength of the hydrogel to address the common problem that most hydrogels are not suitable in surgical sutures (Figure 6) (Kong et al., 2020). Therefore, the research and development of exceptional stromal bio-inks with appropriate printability, senior fidelity, suitable cell viability but low pathological stimulation for 3D bioprinting remains a major challenge (He et al., 2020).

Gelation is one method to modify the physical properties of a candidate material and is a process where (polymerized) molecules are linked tightly, leading to the formation of large, macroscopic molecules if extensive linkages occur (Sun and Tan, 2013). However, the gelation method may harm local tissues and cells, especially with chemical process using (1-ethyl-3-(3-dimethylaminopropyl) carbodiimide) - (N-hydroxy succinimide) (EDC-NHS) system, or with irradiation using UV light (Zhang et al., 2011). After gelation, porosity influences the water content of hydrogel and cell survival (Lai et al., 2013). If porosity is low but pore size is overly large, the construct may have inadequate strength against deformity, but if porosity is high but pore size is too small, only adherence by corneal epithelium and endothelium is possible but spatial fixation of the construct by host keratocyte ingrowth will not occur (Orive et al., 2019). Hence, 3D-printed scaffolds constructed with appropriate and interconnected pores are promising (Ma et al., 2019).

### 7.3 Bioinks for constructing epithelium and endothelium

While, apart from printing main body of the corneal stroma, bio-inks suitable for constructing epithelial or endothelial layers depend less on the biomechanical properties, but more on the biocompatibility as a cell-carrier, therefore possess a wider range of choice. In 2016, Wu Z et al. adopted a hybrid system with the combination of 10% w/v gelatin, 1% w/v alginate, and .513/0.615/0.82/1.025 mg/ml neutralized rat-tail type I collagen solution (Wu et al., 2016). The constructs were subsequently immersed in a 3% calcium chloride solution for chemical crosslinking of sodium alginate at post-printing stage (Wu et al., 2016). In 2018, Sorkio A et al. prepared the epithelial bio-inks using 33% of .1 mg/ml LN521, 50% of defined and serum-free CnT-30 medium with 1 × RevitaCell (Thermo Fisher Scientific, MA, USA), and 17% of 1% w/v hyaluronic acid sodium salt from *Streptococcus* equisaline (MW = 1.5–1.8 × 106 Da) in Tris-buffered saline (Sorkio et al., 2018). Additionally, they made the stromal bio-inks with 44.4% of neutralized human type I collagen, 22.2% of ethylenediaminetetraacetic acid (EDTA) human female AB blood plasma, 22.2% of 40 IU/ml thrombin from human plasma in .1 M tris-buffered saline (TBS), and 11.1% of 10 × Dulbecco’s phosphate buffered saline (DPBS) (Sorkio et al., 2018). In 2022, He B et al. blended 5% GelMA and 20% long-chain PEGDA to form a two-component bio-ink for bi-layer corneal scaffolds (He et al., 2022). These study demonstrates trends for constructing multiple layers with function-specified bio-inks, aiming for anterior lamellar keratoplasty (ALK) or deep lamellar endothelial keratoplasty (DLEK) replacement.

In summary, materials with adequate structural strength were highly anticipated to be ideal bioink for the printing of corneal stromal alternatives to achieve the related biological functions.

## 8 Cell involvement

The cornea is a differentiated and mature organ composed of multiple tissue layers, with each playing an essential part in its physiological function (Pagella et al., 2014). A few researchers have incorporated cellular components to the bioprinted construct, which may aid tissue regeneration and production of ECM for better integration with host corneal tissue or provides a more suitable microenvironment for recruitment of host cells (Sadtler et al., 2016). The involvement of corneal cells recapitulate native physiology of the cornea in the corneal artifact, allowing for regenerative properties of the construct and its ability to respond to environmental stimuli (Xiang et al., 2022). Currently available bioprinting techniques can realise the incorporation of cells into the bioink, yet there are certain limitations present (Xiang et al., 2022). For instance, when using inkjet printing lower cell desity is preferred as high cell density can cause nozzle clogging, which means it is only applicable for corneal stroma that contains low cell density (Xiang et al., 2022). But on the other hand, extrusion-based bioprinting can overcome this limitation as it favours high-viscosity bioinks and thus is friendly with higher cell-density encapsulation (Xiang et al., 2022). Laser polymeration-based technique is flexible in terms of the viscoctuty of bioink, but high laser power is likely to reduce cell viability (Xiang et al., 2022). Hence, DLP-based has an advantage as the lower power of the light source ensures higher cell viability (Xiang et al., 2022).

**FIGURE 5:**
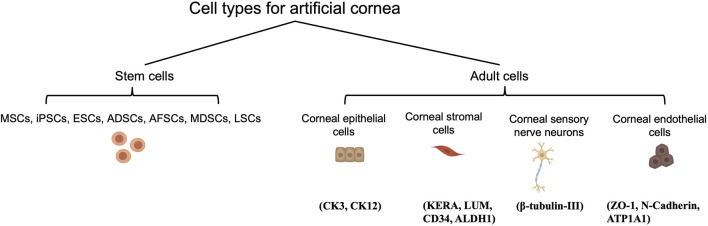
Cell types possible for 3D bioprinting of cornea and their corresponding phenotypic biomarkers.

Cell types possible for 3D bioprinting of cornea and the common marker for each cell types are schematically presented in Figure 5. The mature cornea comprises three cellular layers, i.e., epithelium, stroma, and endothelium, along with two acellular interfaces, i.e., Bowman’s and Descemet’s membranes (Remington and Goodwin, 2011). Together with the regular tear film, these five layers all correlate with the normal cornea’s clarity and two-thirds of the eye’s total refractive power (Thanabalasuriar et al., 2019).

As the cellular interface between tear film and the corneal stroma, the epithelium not only conveys nutrients and oxygen from the corneal surface, but also help maintain an optically smooth surface that is necessary for optimal vision. In addition to the normal tear film, epithelium assist in fighting against corneal infections as a barrier to physical, chemical, and biological agents which may be harmful to the corneal stroma (Zavala et al., 2013). Deficient coverage of a fully functional epithelium on the anterior surface of the cornea issues in epithelial down growth, infection, and extrusion due to stromal melting (Carlsson et al., 2003). Therefore, to minimize these situations, enabling the occurrence and maintenance of a stratified epithelium to cover the keratoprosthesis is the key to its successful development (Ghezzi et al., 2015). The possible benefit from epithelial cells prompted some researchers to incorporate them in their bio-inks. For example, Wu Z et al., in 2016 reported utilizing human corneal epithelial cells (HCECs, RIKEN Biosource Center) for their bioprinted 3D constructs (Wu et al., 2016). Wu Z et al.‘s work marks the first attempt to include epithelial printing into stromal reconstruction. Though potentially beneficial, the advantage of incorporating HCECs in a real-world setting may be limited to trachomatous keratopathy, Stevens-Johnson syndrome, ocular cicatricial pemphigoid, chemical burns, etc., where advanced limbal stem cell deficiency takes place. Instead of focusing on adding epithelial-originated cells, it was suggested that adding patterns to the surface of keratoprosthesis tends to restore functional epithelial coverage more efficiently (Brunette et al., 2017). In 2021, Ulag S et al. reported a novel design of 3D printed PMMA/vancomycin (VAN) scaffolds to treat the Thiel-Behnke corneal dystrophy (Ulag et al., 2021). The scaffold consists of honeycomb structures that would allow better cell adhesion with ameliorated proliferation of cells (Ulag et al., 2021). However, further investigation is required as the antimicrobial activity of scaffold is not able to cover the full device due to undesired permeability of the materials (Ulag et al., 2021). But it is anticipated that the characterized and quantified biochemical and biophysical cues of the anterior basement membrane of the cornea will facilitate epithelial coverage of the implant, as topographic cues impact essential phases of the corneal epithelial wound-healing process, translating into an improved rate of wound healing with subsequent stratification and maintenance of a healthy epithelium (Shen et al., 2017). Therefore, as the corneal epithelium continually regenerates, a synthetic cornea with an external surface which supports the normal growth of autologous epithelial cells would suffice in most situations and minimize the risk of immunological reaction to exogenous epithelial cells (Hunziker et al., 2006).

**FIGURE 6:**
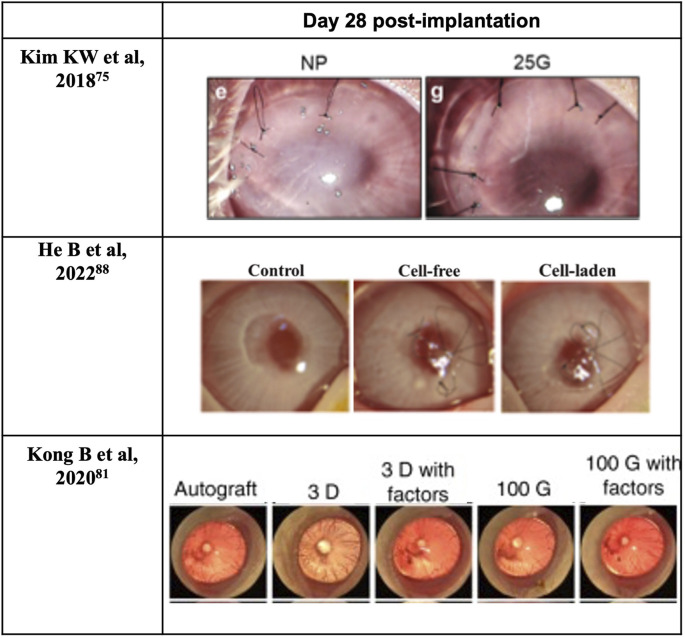
Surgical applications of 3D bioprinted cornea.

Cornea stromal cells, or keratocytes, are normally inactive (quiescent) soon after birth, but are reactivated following corneal insult such as injury or infection (Lwigale, 2015). During intrauterine development and when activated, they can proliferate and synthesize the various components of the ECM, including proteoglycans such as keratan and chondroitin/dermatan sulfate, type I and V collagens, etc. (Lwigale, 2015) Ultimately, the synthesis of proteoglycans and collagens interact and take a crucial part in achieving and maintaining corneal transparency (Lwigale, 2015). Therefore, keratoprostheses of late years center primarily on the bio-integration of the device into the native stroma. Moreover, previous studies have shown that using keratocytes seeded scaffolds can enhance the construct’s mechanical properties (Vrana et al., 2007; Zorlutuna et al., 2007). For instance, the use of cell-laden GelMA hydrogels for 3D bioprinting enhances the biomechanics of the hydrogel construct in comparison with those printed using cell-free GelMA hydrogel (Bektas and Hasirci, 2020). Bektas CK et al. reported that 3D printed hydrogels encapsulated human keratocyte showed a significant post-printing increase of the compressive modulus over 3 weeks, up to 20 kPa and approaching that of the normal human cornea (27–41 kPa), likely from the synthesis of collagens and proteoglycans by keratocytes that was demonstrated with immunocytochemistry (Bektas and Hasirci, 2020). In 2018, Isaacson A and his colleagues isolated human keratocytes from cadaver corneal tissue for use in bioprinted corneal stroma alternatives (Isaacson et al., 2018), which was followed in 2019 by Campos et al. using human keratocytes from donor corneas supplied by the Cornea Bank Aachen for their bioprinted corneal stroma constructs (Campos et al., 2019), and in 2020 by Bektas CK′ and Kutlehria S’s research team using isolated human keratocytes for stroma build-up and high throughput printing (Bektas and Hasirci, 2020; Kutlehria et al., 2020). However, it remains uncertain if the transplanted keratocytes can maintain a long-term pre-fibroblast (dormant) state in such non-physiological environments or corneal scarring would become inevitable and lead to the excessive fibrosis by transdifferentiated myofibroblasts. Interestingly, in 2018, Connon CJ et al. also found an interesting phenomenon that the curvature of the cornea could affect keratocytes (Isaacson et al., 2018). A study in 2019 also pointed out that the alignment of collagen fibrils may be beneficial to cellular activity. In the study, Kim H et al. developed a printing technique with shear induction *via* different diameter of the nozzles and flow rate, of which 25G nozzles showed significantly higher messenger ribonucleic acid (mRNA) levels of keratocyte-specific genes such as KERA and ALDH (Kim et al., 2019). More importantly, the increased cellular activity and bioactivity could even induce further remodeling of the general structure (Kim et al., 2019). A lattice pattern of collagen fibrils that is like native human cornea is found on the printed device after 4 weeks *in vivo* (Figure 6) (Kim et al., 2019). In addition, in 2021, Yao K et al. reported that MSC exosomes could help corneal stroma regenerates better than that of the control group in rat models (Tang et al., 2021). Hence, cytokines/chemokines or spatial features that could help regulate keratocyte activity seems like a superior strategy for stromal regeneration and remodeling than simply adding cells with epithelial-mesenchymal transition (EMT) potentials such as stromal cells or mesenchymal stem cells.

The use of both epithelial cells and keratocytes in 3D bioprinted corneal alternatives has also been reported first in 2018 by Sorkio A et al., using human embryonic stem cells and human adipose stem cells for producing epithelium- and stroma-mimicking constructs, respectively (Sorkio et al., 2018). In their study, they preferred stem-like cells which serve to recreate the genesis of cornea rather than polarized/activated somatic cells like mature keratocytes which easily generate adverse impacts by trans-differentiation into myofibroblasts (Sorkio et al., 2018). Additionally, in 2022, He B et al. conducted bi-layer printing using rabbit corneal epithelial cells for epithelia layer and rabbit adipose-derived mesenchymal stem cells for stromal layer (He et al., 2022). In their study, they proved rabbit corneal epithelial cells’ migration ability on the PEGDA-GelMA hydrogel surface in the *in-vitro* settings (He et al., 2022). However, in the *in-vivo* settings, the fluorescein staining results on the ocular surface under cobalt blue light showed positive signal at the day of keratoplasty indicating remaining epithelial defect even with the 3D printed bi-layer corneal scaffold (He et al., 2022). Therefore, the validity of the laden rabbit corneal epithelial cells as well as rabbit adipose-derived mesenchymal stem cells is with uncertainty.

The very last cell component is corneal endothelial cells. Corneal endothelium is essentially critical in regulating stromal hydration, driven by ionic gradients situated between aqueous humor and stroma (Srinivas, 2010). Sitting between anterior chamber and corneal stroma, corneal endothelium facilitate the transfer of nutrients to and removal of waste and excess water from the stroma, which help maintain its hydration at around 78% (Bonanno, 2003). In addition, endothelial cells produce type VIII collagen, which contributes to the lean assembly of DM (Bonanno, 2003). Endothelial cell loss or cytoskeleton breakdown severely impairs the visual pathway and is irreversible. Although corneal endothelial cells can respond to minor and/or localized losses from trauma or disease by stretching (enlarging) and centripetal migration to the lesions, they usually do not regenerate *in vivo* among primates, including humans (Bonanno, 2003). A minimum cellular density between 400 and 500 cells/mm^2^ is required to prevent stromal over-hydration (resulting in corneal edema) and maintain corneal transparency (Bonanno, 2003). The enlargement of cell size is accompanied by an increase in proportion of cells with variable (non-hexagonal) shape, both of which correlates to compromised endothelial cellular density (Bonanno, 2003). As regeneration of corneal endothelial cells does not normally occur in humans, incorporation of viable endothelium in bioprinted cornea destined for full-thickness keratoplasty may be required, depending on the water-retaining capability of the bio-inks used. Of late years, corneal endothelial sheet engineering has emerged as an innovative scaffold-based alternative to corneal transplant by means of the *in-vitro* cultivation of human corneal endothelial cells (Khalili et al., 2021; Parekh et al., 2021). Meanwhile, cell sheet-based bio-ink has proved its efficacy in shape fidelity, reproducibility, and automated deposition and can be applied to scaffold-free inkjet-based 3D bioprinting (Bakirci et al., 2017). What’s more, in severe cases of corneal injuries that affect the regenerative power of the tissue, regenerative medicine for the cornea such as cell-based therapies or treatment with cytokine/growth factor cocktails are expected to re-establish the microenvironment of the ocular surface (Yazdanpanah et al., 2019). Up to now, bioprinting with corneal endothelial cells has rarely been attempted. Three mechanisms that contribute to endothelial cell-cycle arrest in the G1-phase of mitosis upon full development are: i) Inhibition of cellular contact; ii) Absence of bio-molecular stimulation; iii) TGF-β2 suppression of S-phase (Bonanno, 2003). In addition to the non-proliferative property, the quality of the donated endothelium is susceptible to age, cellularity, donor death-to-preservation period, the overall health of the donor, and the specific cause of death (Schaub et al., 2017). Based on this, in 2013, human corneal endothelial cells *ex vivo* models were reported covering central and peripheral zones of young or adult donor corneal endothelium with the mechanism of releasing cellular junctions but preserving the presence of efficient growth factors. The combining utility of insulin and bFGF promoted mitosis in peripheral corneal endothelial cells. Meanwhile, nerve growth factor (NGF), bovine pituitary extract, and EGF were applied to support the expansion of central endothelial cells (Guérin et al., 2021). In 2018, Kim KW and his colleagues reported to have built endothelial layer with RNase 5 vector‐transfected human corneal endothelial cells at first passage (Kim et al., 2018). The use of RNase 5 grafted materials gave superb performance in terms of recovery after surgery. *In vivo* study conducted by Kim KW showed the corneal clarity with RNase 5 grafted groups was close to the healthy cornea while the control group remained at low clarity (Kim et al., 2018). Stem cells have enormous potential in regenerative medicine as they can differentiate into cells of multiple lineages including corneal limbal cells, epithelium, stroma, or endothelium (Hsu et al., 2015). It is thus meaningful to demonstrate the possibility and necessity of adding stem cell into the bio-inks for an improved regenerative capability in the artificial corneal construct (Ong et al., 2018). Thus, with the help of stem cell differentiation, differentiated corneal endothelial cells are expected to address this issue.

Taken together, the cell therapy approach includes propagating healthy human corneal cells whose physiological phenotypes were serviced for replacing diseased corneal layers (Shen et al., 2018). More and more research groups tend to design and bioprint multi-functional layers, mostly stromal and epithelial layers, but the materials, cell components and the printing techniques required vary widely for each layer, which needs to be accounted for in the preprinting planning process. However, many autologous or primary cell types are difficult to isolate and culture *in vitro*, and are limited by a finite lifespan (Ong et al., 2018). For example, isolated primary corneal epithelial cells grows relatively slow, with a tendacy to differentiate into fibroblast in stressed condition. In addition, not all cellular proliferation processes are desirable, mesenchymal stem cells and keratocytes have the potential of transdifferentiation into myofibroblasts, and its proliferation can be detrimental for a bioprinted corneal construct due to likely reduction in corneal transparency from stromal fibrosis. Moreover, it is challenging for sterile storage of the cell/growth factor-laden corneal scaffolds especially for a long-term, and maintenance of cellular phenotypic characteristics and viability during long-distance transportation (Huang and Li, 2007). Besides replicating the biological function of the native tissue, it may be desirable for host native cells to be able to integrate into the printed scaffold, yet not so excessive as to compromise corneal clarity and homeostasis. Therefore, effectively including cellular components into bioprinted corneal constitutes is worthy of further exploration.

## 9 Keratoprothesis and 3D-bioprinted cornea

Tissue-engineered cornea initially came up to address the various complications of keratoprosthesis including the complexity of the transplantation processes and limited visual field (Zhang et al., 2019b). Aiming for higher proximity to a natural cornea, 3D bioprinting, with its proved feasibility in many fields of tissue engineering such as skin, cardiac muscle, and oral and maxillofacial tissue, has become a new two for corneal substitutes. Nevertheless, as 3D bioprinting is still in the early stage of research and development, many aspects in generating the 3D bio-printed cornea awaits to be optimized, such as improved the biomechanical properties and transparency of artificial corneas (Trampe et al., 2018). There is still a long way to go to transfer the artificial cornea from experimental stage to clinical trials, where both the refinement of 3D bioprinting techniques, including the use of composite printing technology and bioinks with improved performance, and a more precise understanding of corneal anatomy, physiology and mechanical properties are needed. Meanwhile, a comprehensive international standard with clear illustration of the biological, mechanical and optical properties required for the 3D bio-printed artificial cornea must be introduced to facilitate the construction of a fully functional product of artificial cornea which can be approved in clinics (Zhang et al., 2019b).

## 10 Conclusion and future prospects

This review examined all the essential elements in relation to 3D printing of keratoprosthesis and discusses the current advances, limitations and expectations. Concerning the bioink, the combination of hydrogels with scaffolds made of both optical transparency and stable structural strength materials have the potential to balance the mechanical properties and biocompatibility which are both required. Meanwhile, the inclusion of stem cells into the bioprinted corneal construct is promising upon further research for fabricating a corneal constitute with greater regenerative capability. Concerning bioprinting techniques, DLP, with its capacity to address the limitations of other tecniques particularly in terms of ensuring cell viability and its high-thoughput nature, serves as a promising choice for efficiently fabricating artificial corneas in future (Xiang et al., 2022). Meanwhile, its compatibility with transparent and hydrophilic biomaterials, and high precision fabrication also makes it most ideal for maintening the optical properties of the bioprinted corneal tissue (Xiang et al., 2022). Although 3D bioprinted cornea is not yet approved in clinical application, some clinics have used 3D printed personalized corneal models during consultation (Velázquez et al., 2020). Feedback from patients were highly positive as 73.8% of the patients reported the use of 3D model had helped them to understand their diesases better (Velázquez et al., 2020). In fact, the use of 3D printing is not only limited in treating disease but also important to clinical services.

**FIGURE 7:**
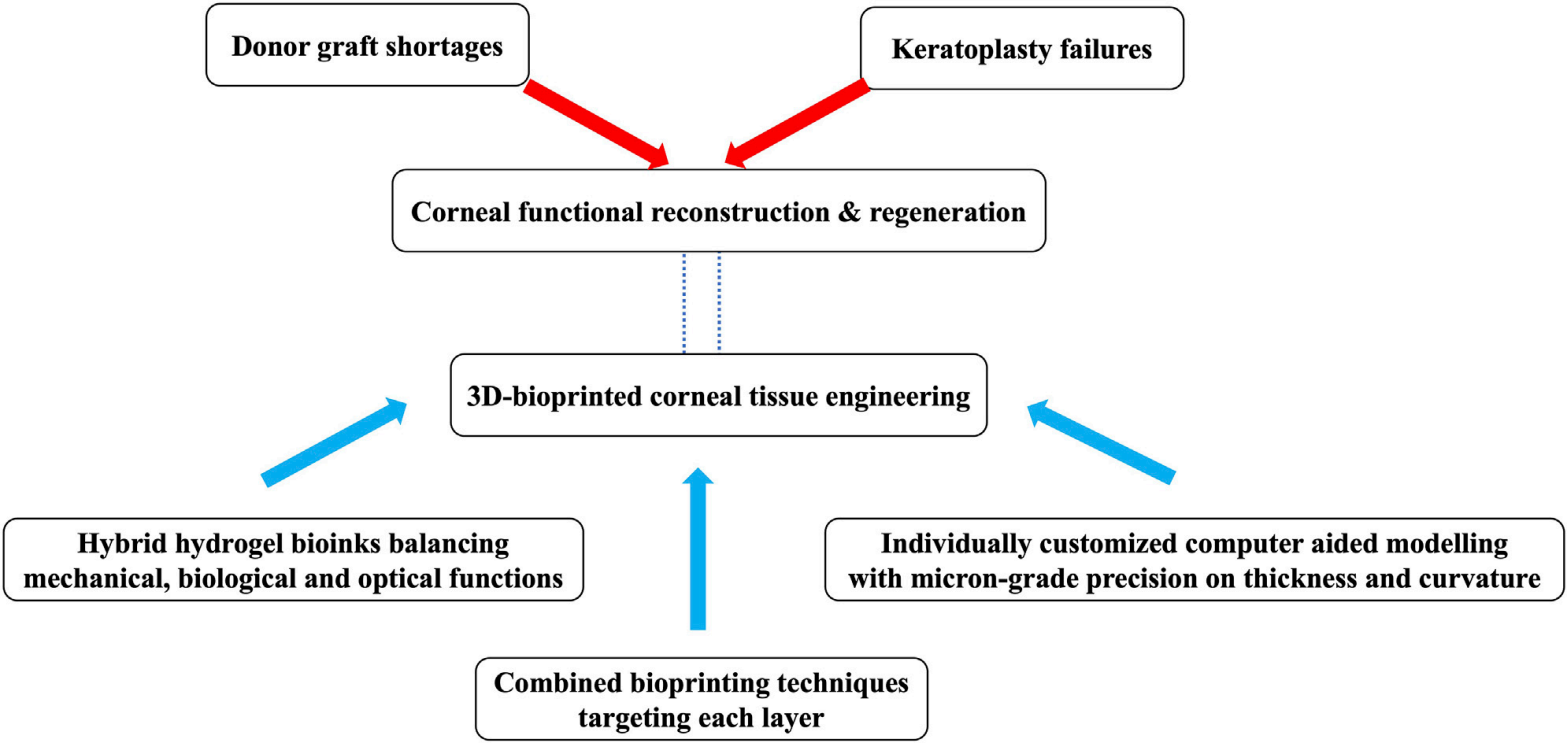
Take-home schematic summary.

Although much progress has been made in 3D bioprinting related research, data from clinical trials is lacking and the complication of printed grafts remains unclear. Considering the complexity and uncertain clinical performance of bioprinted corneal device which requires a combination of several modern technologies including 3D printing and stem cells technology, it may be difficult to recruit human subjects. Longterm biocompatibility and host reaction will also need to be addressed before these devices can be safely adopted for clinical use. Despite the lack of clinical data, current studies have demonstrated low toxicity and good mechanical strength of 3D bioprinted devices. As summarized in Figure 7, it seems likely that a biologically and physiochemically safe, and patient-specific corneal alternative produced by 3D bioprinting can be achieved in the near future given the rate of current progress, and this will help alleviate the current worldwide shortage of donor corneas for the treatment of corneal blindness.
